# Synthesis of NiO Nanotubes via a Dynamic Thermal Oxidation Process

**DOI:** 10.3390/ma12050805

**Published:** 2019-03-08

**Authors:** Wenfeng Xiang, Zibin Dong, Yi Luo, Jiali Zhao, Jia-ou Wang, Kurash Ibrahim, Haihong Zhan, Wenzheng Yue, Haizhong Guo

**Affiliations:** 1 State Key Laboratory of Petroleum Resources and Prospecting, China University of Petroleum, Beijing 102249, China; wfxiang@cup.edu.cn (W.X.); 11406605@163.com (Z.D.); wyue@163.com (W.Y.); 2 School of Physical Engineering, Zhengzhou University, Zhengzhou 450001, China; luoyilogic@163.com; 3 Beijing Synchrotron Radiation Facility Institute of High Energy Physics, Chinese Academy of Sciences, Beijing 100049, China; zhaojiali@ihep.ac.cn (J.Z.); jowang@ihep.ac.cn (J.-o.W.); kibrahim@ihep.ac.cn (K.I.); 4 Department of Biochemistry, Pingdingshan Vocational and Technical College, Pingdingshan 467000, China; hhzhan@163.com

**Keywords:** NiO nanotubes, thermal oxidation, microstructure, critical transition temperature

## Abstract

Nickel oxide (NiO) nanotubes were synthesized via a thermal oxidation process from Ni nanowires. The effects of oxidation temperature on the morphology, microstructures, and composition of nanowires were investigated using scanning electron microscopy, X-ray diffraction, and X-ray photoelectron spectroscopy. The results show that the Ni nanowires convert initially to Ni/NiO core-shell nanowires with increasing annealing temperatures, and then to the nanotubes at the critical transition temperature of about 425 °C. Our findings provide useful information for the preparation of NiO nanotubes to meet the required applications.

## 1. Introduction

Nickel oxide (NiO) nanostructures are currently being exploited as active components in a wide range of applications including photocatalysis [1], solar energy cell [2,3], photovoltaics [4], electrochemical supercapacitors [5], and sensors [6] for their excellent electronic, photonic, catalytic, and gas-sensitive properties. Much effort has been devoted to the synthesis of different NiO nanostructures, such as wires [7], rods [8], particles [9], flowers [10], sheets [11], and core-shell structures [4]. Unlike nanowires (NWs) and nanoparticles, it is difficult to synthesize NiO nanotubes (NTs) because of the hollow structures in nanorods and nanowires.

Reports on large scale synthesis of NiO NTs are quite rare. Gao et al. [12] prepared the NiO NTs via aluminium oxide templates. However, NTs have lots of particles. Tian et al. reported a facile ZnO template-assisted hydrolysis method to fabricate aligned NiO NTs [13]. Using filter paper as the template, hollow NiO NTs were prepared via a bio-template engaged route [14]. In order to enhance the gas sensing performance, Pt-functionalized NiO NTs were synthesized by an electrospinning method [15]. Compared with the above methods, the simplest method of fabricating NiO NTs is from through direct thermal oxidation of Ni NWs. Ni/NiO core-shell NWs are usually formed by thermal oxidation of Ni NWs [16]. With increasing temperatures, NiO NTs were formed by Ni atoms diffusing from the interior to the exterior of Ni NWs. However, an in-depth understanding of the transformation process from NWs to NTs is still unclear, and further investigation is highly desirable.

In this study, NiO NTs were synthesized by the thermal oxidation of Ni NWs. The annealing temperature effects on the morphology, microstructures, and composition of nanostructures were investigated, and the transformation temperature of NTs formation from NWs was confirmed.

## 2. Experimental

Ni NWs were synthesized by the chemical reduction method, and details of the synthesis process are described elsewhere [17,18]. After preparation, Ni NWs were obtained by filtering, washing for three times with distilled water and ethanol sequentially, and drying in a 60 °C oven for 12 h. The NiO NTs were prepared by simple thermal oxidation of these Ni NWs. Because the annealing temperature plays an important role on the formation of NiO NTs, a series of experiments was conducted in a tubular furnace under the different oxidation temperatures from 200 to 600 °C for 2 h to investigate the effect of the oxidation temperature on NTs formation. The crystalline structure and composition were characterized by X-ray diffraction (XRD, Bruker, Billerica, MA, USA). The size and surface morphology of NWs were characterized by scanning electron microscopy (SEM, Hitachi SU8010, Tokyo, Japan). X-ray photoelectron spectroscopy (XPS) was obtained at the Beijing Synchrotron Radiation Facility of the Chinese Academy of Sciences (Beijing, China). The oxidation process was monitored in-situ using a Pyris 1 thermogravimetric analyzer (TGA; PerkinElmer, Waltham, MA, USA).

## 3. Results and discussion 

**Figure 1:**
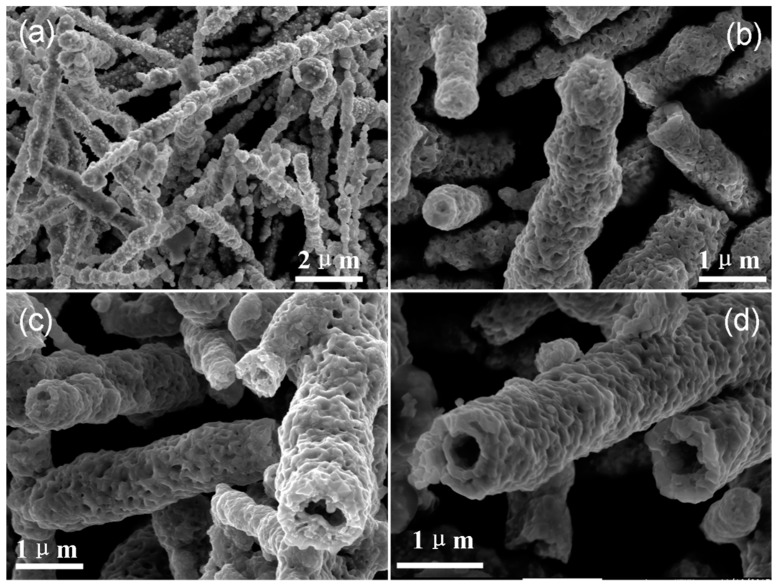
SEM images of (**a**) as-prepared Ni NWs, (**b**–**d**) Ni NWs annealed at 400, 500, and 600 °C, respectively.

Figure 1 shows SEM images of Ni NWs annealed at different temperatures. Figure 1a exhibits the SEM image of the as-prepared Ni NWs, and it can be seen from Figure 1a that many aciculas cover the surface of Ni NWs. The SEM image of Ni NWs annealed at 400 °C for 2 h is shown in Figure 1b. It can be found that all the aciculas disappeared and the surface roughness of NWs decreased with the increase in oxidation temperature, moreover, a small cavity exists in the end of NWs. When the oxidation temperature increased to 500 °C, the wire-like structures transformed into tube-like structures and NTs were obtained. Complete NT structures were evidently observed from the samples prepared at an annealing temperature over 600 °C. 

**Figure 2:**
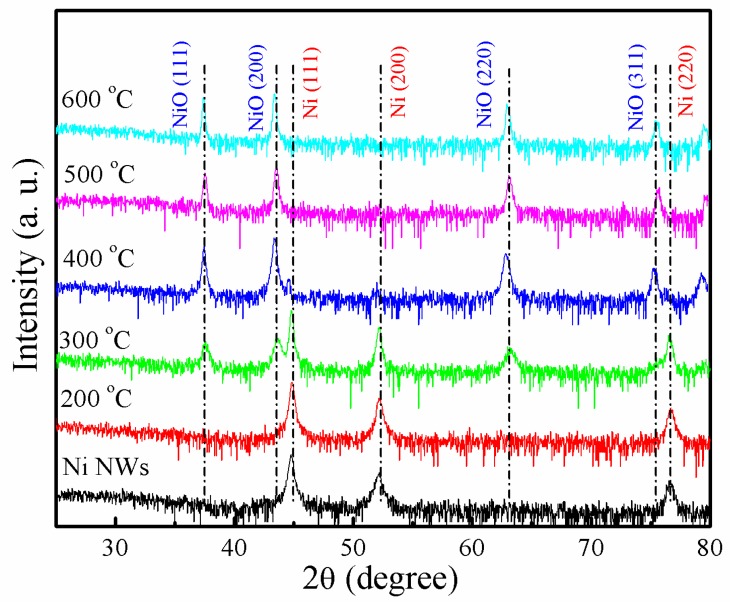
XRD pattern of Ni NWs as a function of oxidation temperature.

The corresponding XRD patterns of Ni NWs oxidized at different temperatures are shown in Figure 2. All the diffraction peaks in the XRD pattern can be well indexed to metallic Ni (JCPDS No. 04-0850) and NiO (JCPDS No.47-1049). When the annealing temperature was blow 200 °C, no diffraction peaks from NiO were observed, except Ni (111), (200), and (220) peaks. The NiO and Ni diffraction peaks coexisted when the temperature was above 300 °C. It is indicated that Ni/NiO core-shell NWs were formed. Moreover, the density of Ni diffraction peaks gradually decreased with increasing annealing temperatures. For the sample annealed at 500 °C for 2 h, the Ni diffraction peaks totally disappeared. 

**Figure 3:**
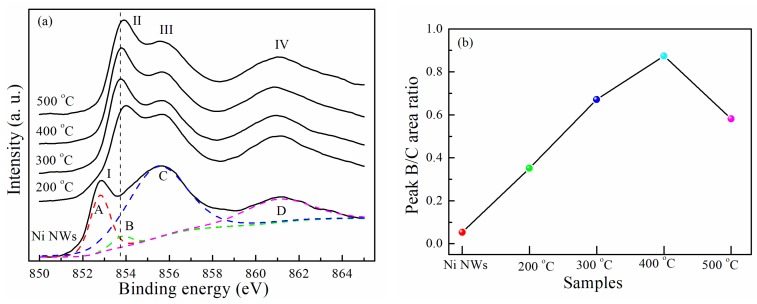
Ni 2p_3/2_ XPS spectra (**a**) and peak B/C area ratio (**b**) as a function of oxidation temperature.

Figure 3a shows the Ni 2p_3/2_ XPS spectra of Ni NWs as a function of the oxidation temperature. The spectra are similar and present almost the same structures except the spectrum of the as-prepared Ni NWs with peak I. The spectra in Figure 3a were fitted using four peaks. Peak A (852.8 eV), peak B (853.8 eV), and peak C correspond to the binding states of metal Ni, Ni^2+^, and Ni^3+^, respectively. The main difference between these spectra was that peak II position shifted with the oxidation temperature. Ni NWs had no peak II in the spectrum. Peak II of Ni NWs that annealed at 200 °C existed, and the position was higher than that of peak B for NiO, indicating that Ni NWs were oxidized. As the oxidation temperature increased, the peak II shifted slightly towards the lower binding energy side. The positions of Ni NWs’ peak II oxidized at 300 and 400 °C was similar to that of peak B. It is interesting to note that peak II of Ni NWs which annealed at 500 °C, shifted back to the higher binding energy side. Figure 3b shows the area ratio of peak B to peak C. It can be seen that the content of NiO increases with annealing temperature increase from 200 to 400 °C, i.e., thickness of the NiO layer on the surface of NWs increases. However, the area ratio decreased at 500 °C annealing temperature. It is indicated that the intensity of Ni^3+^ cations increases in NWs and NT structures with the opening end forms. It can be concluded that the transformation temperature from NW to NT structures is between 400 and 500 °C.

**Figure 4:**
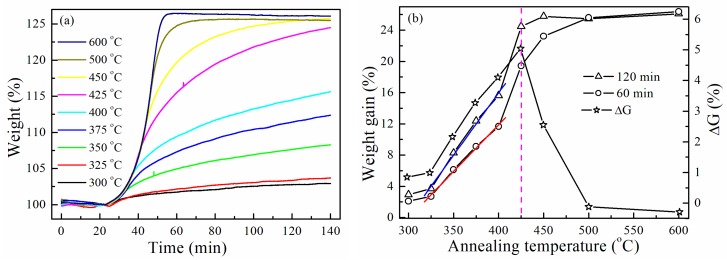
TGA curves of Ni NWs (**a**) and variation of weight gain and ∆G (**b**) as a function of oxidation temperature.

In order to shed more lights on the effect of temperature on NTs formation, the oxidation process of Ni NWs was investigated in the temperature range between 300 and 600 °C using TGA (Figure 4a). Weight gain continuously increased during the oxidation process when the oxidation temperature was below 450 °C, and the oxidation reaction time was less than 40 min at the oxidation temperature above 500 °C. Figure 4b shows the weight gain of Ni NWs after oxidation for 60 and 120 min, respectively. It is interesting to note that the weight of NWs increases linearly with temperature increase from 325 to 400 °C, and the weight gains were ~3–4 % every 25 °C. However, the maximum weight gains were 7.8 % and 8.9% when the temperature increased from 400 to 425 °C, respectively. ∆G is the difference of weight gain for Ni NWs after oxidation for 120 and 60 min. As shown in Figure 4b, a ∆G peak was found, and the peak position was located at 425 °C. These results suggest that a distinctive change in Ni NWs’ weight at the oxidation temperature of 425 °C is observed.

The transformation from Ni NWs to NiO NTs resulted from solid-gas reactions involving the diffusion of reactants. During the first step, oxygen atoms in the air were adsorbed onto the outer surfaces of Ni NWs and reacted with Ni to produce a NiO layer. With the continuous adsorption of oxygen atoms onto the surface, electrons can pass rapidly through the oxide by tunneling to establish equilibrium between the metal and adsorbed oxygen. This process creates an electronic field in the thin oxide layer capable of pulling metal and oxygen ions through the oxide film, inducing the increase in thickness of the NiO layer. Owing to the diffusion of oxygen atoms from the outside to the inside, NiO*_x_* (*x* ≤ 1) is formed between the NiO shell and metal Ni core. Simultaneously, the diffusion of Ni ions and O ions was hindered by the NiO layer. Moreover, thickness of the NiO barrier layer increased with increasing annealing temperatures [16]. This is due to the fact that the formation of NiO on the surface of Ni NW is dominated by an outward diffusion of Ni. Thickness of the NiO layer and the amount of outward-diffused Ni captions increased with increasing annealing temperatures. When the annealing temperature increased to a certain value, hollow structures were formed in NWs. Based on the SEM, XPS, and TGA results, our observations suggest that the transformation temperature from Ni NWs to NiO NTs is ~425 °C. Under higher oxidation temperatures, the diffusion energy of Ni atoms will be larger than the barrier energy of the NiO layer. Therefore, hollow structures will be enlarged. Pure NTs were fully formed at 600 °C (shown in Figure 1d).

## 4. Conclusions

NiO NTs were synthesized by a thermal oxidation process from Ni NWs. Firstly, the Ni/NiO core-shell NWs forms at temperatures blow 400 °C. The hollow structures in NWs occur at 500 °C and pure NTs were fully formed at 600 °C. XPS results showed that the content ratio of NiO/Ni_2_O_3_ increases with increasing temperature. However, the content ratio decreases when the temperature rises to 500 °C. When the temperature increases from 400 to 425 °C, the NWs have the largest weight change. Compared to the weight change of Ni NWs after oxidation for 120 and 60 min, the largest weight change was observed for Ni NWs annealed at 425 °C. It is found that the transformation temperature from NW structures to NT structures is ~425 °C.
